# Dietary antioxidant intake increases ankle brachial pressure index in men but not in women: a cross-sectional study

**DOI:** 10.3389/fcvm.2024.1343135

**Published:** 2024-02-08

**Authors:** Yuting Wang, Jianfeng Wang

**Affiliations:** ^1^ Intensive Care Unit, Zhongshan Hospital (Xiamen), Fudan University, Xiamen, China; ^2^ Department of Dermatology, The First Hospital in Quanzhou Affiliated to Fujian Medical University, Quanzhou, China

**Keywords:** ABPI, CDAI, antioxidant, NHANES, mortality

## Abstract

**Objective:**

Atherosclerosis is a significant cause of cardiovascular and cerebrovascular diseases, with a greater impact on men than women. Dietary antioxidant intake is inversely related to the risk of atherosclerosis development. We aimed to investigate the association between dietary composite antioxidant intake and the ankle brachial pressure index (ABPI). The ABPI is not only used for assessing the progression of arterial lesions but also for stratifying the risk of atherosclerotic disease.

**Methods:**

We conducted a cross-sectional analysis involving 1,049 participants from the National Health and Nutrition Examination Survey (NHANES). We examined six antioxidants (zinc, selenium, carotenoids, and vitamins A, C, and E) and a composite dietary antioxidant index (CDAI) derived from these antioxidants as exposure variables. The primary outcomes encompassed cardio-metabolic parameters, including body mass index (BMI), body fat mass (BFM), systolic and diastolic blood pressure, triglycerides, HDL and LDL cholesterol, C-reactive protein, and the Ankle-Brachial Pressure Index (ABPI). Associations and interactions between variables were assessed using linear regression analyses. Moreover, mediation and moderation analysis is employed.

**Results:**

Hierarchical multiple regression analysis revealed that among men, dietary intake of zinc, selenium, and vitamin A remained positively associated with a higher ABPI even after adjusting for covariates. Conversely, in the stratified regression analysis based on CDAI quartiles, a U-shaped association between CDAI and ABPI was suggested. Notably, no significant association between dietary antioxidant intake and ABPI was observed among women. CDAI, intake of Vitamin A, Vitamin C, and Vitamin E do not influence all-cause death through mediation by abpi, but rather have a direct effect on all-cause death. Moreover, there is a significant interaction between the intake of Vitamin A and gender, where a daily intake of Vitamin A more than 776 ug is especially beneficial for women.

**Conclusion:**

The combined intake of nutrients with antioxidant properties may prevent the initiation and progression of atherosclerosis and influence the outcome in a sex-specific manner.

## 1 Introduction

Ankle-Brachial Pressure Index (ABPI), a non-invasive diagnostic tool, measures the ratio of systolic blood pressure at the ankle to that at the brachial artery in the arm, serving as an indicator of peripheral artery disease (PAD) and vascular health (1). Antioxidants, encompassing an array of compounds such as vitamins, minerals, and phytochemicals, play a crucial role in counteracting oxidative stress, a process implicated in various chronic diseases (2). The ABPI and the intake of antioxidants have both emerged as influential factors in cardiovascular health, with recent research highlighting the importance of considering sex-specific differences in their association.

Sex-specific differences in cardiovascular health have gained prominence, underscoring the need to explore the interplay between ABPI and antioxidant intake in a nuanced manner. Cardiovascular diseases often manifest differently between males and females, with variations in risk factors, disease prevalence, and outcomes (3, 4). Factors like hormonal differences, genetics, and lifestyle choices contribute to these disparities, motivating the investigation of sex-specific associations between ABPI and antioxidant intake. The ABPI serves as a valuable marker of arterial stiffness and peripheral vascular function. Sex-specific variations in vascular health are well-documented, with differences in arterial compliance and endothelial function between men and women (5). These disparities could potentially influence the relationship between ABPI and antioxidant intake. Moreover, oxidative stress, a driving force behind cardiovascular diseases, may be influenced by sex-related factors, leading to divergent responses to antioxidants. Antioxidants have been extensively studied for their ability to combat oxidative stress and mitigate the risk of chronic diseases. Their impact on cardiovascular health, however, might differ between sexes due to varying physiological and hormonal profiles. This raises intriguing questions about the potential modulatory effects of antioxidant intake on ABPI in a sex-specific context.

This study seeks to provide an in-depth exploration of the association between the ABPI and antioxidant intake, emphasizing the importance of considering sex-specific differences. We aim to elucidate how sex-related factors might influence the relationship between ABPI and antioxidants, shedding light on potential sex-specific interventions and contributing to a more tailored approach to cardiovascular health management.

## 2 Methods

### 2.1 Study population

**Figure 1:**
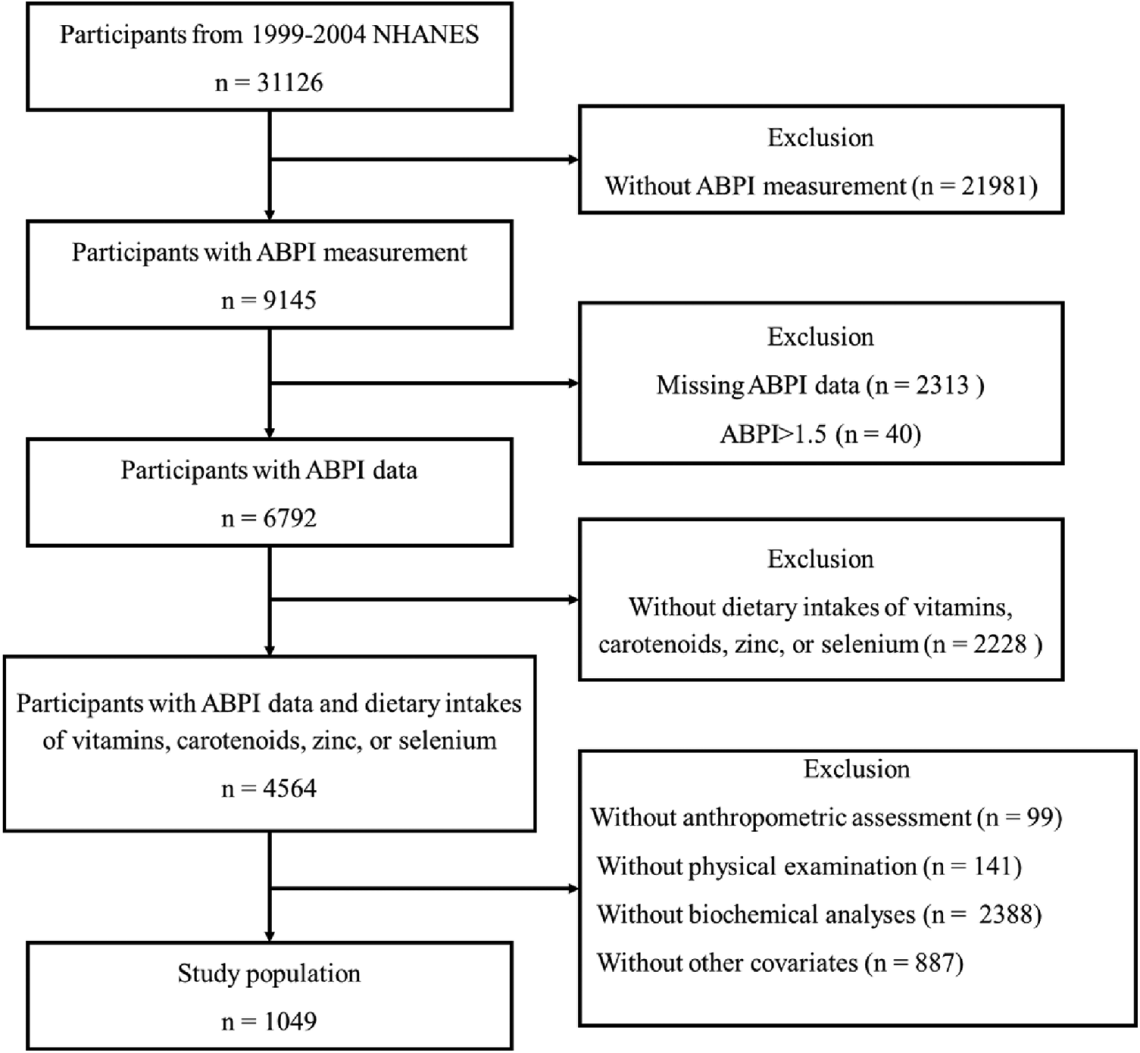
Illustration of inclusion and exclusion criteria as utilized to select the final study population.

The National Health and Nutrition Examination Survey (NHANES) is a cornerstone of public health research and epidemiological studies conducted by the Centers for Disease Control and Prevention (CDC) in the United States. The NHANES data are released every 2 years. Data from the 1999–2004 NHANES cycle were used in this analysis, because these cycle speciﬁcally provided data for ABPI. All participants aged 40 years and over were eligible, as participants aged 40 years and older were asked to undergo the ABPI measurement. Participants were excluded if (1) Their dietary intakes of vitamins, carotenoids, zinc, or selenium are missing; (2) Their ABPI data are missing or ABPI > 1.5, which is considered an outlier (6, 7); (3) Their data for anthropometric measurements, including height, weight, and body fat mass (BFM), are missing; (4) Their data from physical examinations and biochemical analyses, including blood pressure (BP), total cholesterol, low-density lipoprotein cholesterol (LDL-cholesterol), high-density lipoprotein cholesterol (HDL-cholesterol), triglycerides, glycated hemoglobin (HbA1c), glucose, creatinine, urea nitrogen, and C-reactive protein (CRP), are missing; (5) The available information does not encompass their socio-demographic characteristics (such as age, gender, education level, and income), lifestyle factors (such as smoking habits, alcohol consumption, and physical activity), or medical records related to diabetes, hypertension, and hyperlipidemia. Figure 1 illustrated the inclusion and exclusion criteria employed in the selection of the final study population (Figure 1). The protocol was approved by the National Center for Health Statistics Institutional Review Board, and all participants provided written informed consent.

### 2.2 Dietary assessment

Dietary intake reports of six antioxidants (zinc, selenium, carotenoids and vitamins A, C and E), and total energy intake were included as exposures. All participants in the NHANES study are eligible for two 24-hour dietary recall interviews (8). The initial dietary recall interview is conducted face-to-face within the Mobile Examination Center, while the subsequent interview is conducted via telephone within a span of 3–10 days. In this analysis, dietary intakes of six antioxidants and total energy intake were assessed using 1-day values for individuals with single recalls and 2-day means for others. To comprehensively assess the synergistic impact of dietary antioxidants on ABPI, we employed a modified rendition of the Composite Dietary Antioxidant Index (CDAI), originally devised by Wright et al. (9).

### 2.3 ABPI measurement

Participants assume a supine position on the examination table for the assessment. Systolic pressure measurements are taken on the right arm (brachial artery) as well as on both ankles (posterior tibial arteries). If a participant has conditions such as a rash, an open wound on the right arm, a dialysis shunt, or a history of right-sided radical mastectomy that could compromise accurate measurement or lead to discomfort, the brachial pressure measurement is conducted using the left arm. Systolic blood pressure is recorded twice at each site for participants aged 40–59 years, while those aged 60 years and older undergo one measurement at each site. The computer system automatically calculates the Ankle-Brachial Pressure Index (ABPI). Specifically, the right ABPI is determined by dividing the mean systolic blood pressure in the right ankle by the average blood pressure in the arm. Similarly, the left ABPI is calculated by dividing the mean systolic blood pressure in the left ankle by the arm's mean blood pressure. The mean blood pressure values for both the arm and ankles are derived from the first and second readings taken at each respective site. For individuals aged 60 years and older, since the second reading is absent, the mean values represent the initial recorded blood pressure reading at the given site. This also applies to individuals aged 40–59 years who have a missing value for either the first or second blood pressure reading. In this analysis, the lower ABPI value from both the left and right sides is regarded as the definitive ABPI. If the calculated ABPI ≥ 1.3, the higher of the two values is chosen.

### 2.4 Anthropometric assessment

BMI was calculated as weight in kilograms divided by height in meters squared. Body fat mass was computed employing prediction equations derived from the NHANES survey, encompassing 7,531 men and 6,534 women who underwent dual-energy x-ray absorptiometry (DXA) examination, as formulated by Lee DH et al. (10).

### 2.5 Physical examination and biochemical analyses

Blood pressure (BP) was taken on all eligible individuals using a mercury sphygmomanometer. The technique used to obtain BP follows the recommendations of American Heart Association Human Blood Pressure Determination by sphygmomanometers (11).

Biochemical analyses were performed with a Hitachi Model 704 multichannel analyzer (Boehringer Mannheim Diagnostics, Indianapolis, IN), which encompassed the measurement of glucose, total cholesterol, triglycerides, creatinine, and urea nitrogen. HbA1c was measured by a fully automated glycohemoglobin analyzer, utilizing the principle of boronated affinity high performance liquid chromatography (HPLC) (Primus Corporation). HDL-cholesterol was measured using a direct immunoassay technique the LDL-cholesterol level was calculated according to the Friedewald equation. Latex-enhanced nephelometry is utilized for quantifying CRP.

### 2.6 Other covariates

Socio-economic features (such as age, gender, educational attainment, and family income to poverty ratio, PIR), lifestyle behaviors (including smoking habits, alcohol consumption, and level of physical exercise) and medical background of diabetes, hypertension, and hyperlipidemia were obtained through personal interviews (12). The degree of physical activity was assessed using the Czech adaptation of the International Physical Activity Questionnaire long form (IPAQ-L) and quantified as MET-min/week (13). Smoking status was classified into three categories: “never” (individuals who had smoked fewer than 100 cigarettes in their lifetime), “former” (individuals who had smoked more than 100 cigarettes in their lifetime but currently do not smoke at all), and “current” (individuals who had smoked more than 100 cigarettes in their lifetime and currently smoke either some days or every day). Alcohol consumption was classified into five categories: “never” (had <12 drinks per week in lifetime, one standard drink contains 14 grams of pure alcohol), “former” (had ≥12 drinks in 1 year and did not drink last year, or did not drink last year but drank ≥12 drinks in lifetime), “mild” (1 drink per day for female and 2 drinks per day for male), “moderate” (2 drinks per day for female and 3 drinks per day for male; or binge ≥2 and binge <5 per month), “heavy” (3 drinks per day for female and 4 drinks per for male; or binge ≥5 per month). Hypertension was characterized by a blood pressure measurement of 140/90 mmHg or higher, a previous diagnosis of hypertension, or the use of antihypertensive medication. Hyperlipidemia was determined by the presence of total cholesterol levels equal to or exceeding 5.0 mmol/L, LDL-cholesterol levels equal to or exceeding 3 mmol/L, triglyceride levels equal to or exceeding 1.7 mmol/L, or the use of lipid-lowering medication. Diabetes mellitus was identified by a previous diagnosis of diabetes, a fasting glucose level equal to or exceeding 7 mmol/L, or the use of antidiabetic medication.

### 2.7 Outcomes

All-cause mortality, referring to death from any cause until 31 December 2018, was the primary outcome. Mortality data were extracted from the 1999–2004 NHANES public-use linked mortality files. We analyzed the period from enrollment, marked by the date of the interview, to mortality for the purpose of censoring. The International Classiﬁcation of Diseases, Tenth Revision codes (I00-I09, I11, I13, I20-I51, I60-I69) were used to deﬁne cardiovascular deaths. Any participant not matched with any death records was considered alive throughout the follow-up period.

### 2.8 Statistics

The characteristics of the study participants were depicted using percentages (%) or median along with interquartile range (IQR). Prior to subsequent analyses, the normal distribution of continuous variables was examined using the Kolmogorov–Smirnov test. For continuous variables exhibiting skewed distribution, the Mann–Whitney *U*-test (for comparisons between two groups) or the Kruskal–Wallis test (for comparisons involving three or more groups) was utilized. The *χ*^2^ test was employed for contrasting categorical variables. Spearman tests were carried out for correlation analyses. To explore the relationships between dietary antioxidant intakes and ABPI, linear regression models were employed. Age, previous medical history, diabetes, hypertension, hyperlipidemia, income, education, smoking, alcohol consumption, physical activity, and total calorie intake were considered in Model 1. Model 2 included additional adjustments for body mass index (BMI), body fat mass (BFM), blood pressure (both systolic and diastolic), triglycerides, LDL-cholesterol, HDL-cholesterol, and CRP. Furthermore, we examined potential gender-related interactions in the relationship between antioxidant intake, CDAI, and ABPI. For female participants, an extra adjustment was made in Model 3 to account for menopause status. To address multiple comparisons, the Bonferroni method was employed, and adjusted *p*-values were obtained by multiplying the original *p*-value by the number of comparisons. The relationships among antioxidant intake, CDAI, ABPI, gender, and outcomes are examined through mediation and moderation effect analyses. The risk of outcomes occurrence influenced by various factors is analyzed using Cox regression analysis. All statistical tests were performed as two-tailed tests, and a *p*-value less than 0.05 was considered statistically significant. SPSS software (version 24.0) was employed for all statistical analyses.

## 3 Results

The study included 1,049 individuals (median age 57.00 years, IQR = 20.00; 53.48% of men), who satisfied the inclusion and exclusion criteria, with 46.52% being females and 67.83% being menopausal women (Table 1). While women exhibited a lower BMI (*p* = 0.001), men displayed a lower body fat mass (*p* < 0.001). In addition, men reported higher glucose and HbA1c levels compared to women (*p* < 0.001 and *p* = 0.006, respectively), and a greater prevalence of previous diabetes history (*p* = 0.003). Men also showed higher levels of creatinine (*p* < 0.001), urea nitrogen (*p* < 0.001), and triglycerides (*p* = 0.009), as well as lower levels of HDL-cholesterol (*p* < 0.001) than women. Age, educational level, PIR, use of lipid-lowering or antihypertensive medications did not differ between the two groups.

**Table 1:** Characteristics of study participants in general and stratified by gender.

Characteristics median (IQR) or %	Total (*N* = 1,049)	Male (*N* = 561)	Female (*N* = 488)	Z/*χ*^2^	*p*-value
Age, year	57.00 (20.00)	57.00 (21.00)	58.00 (20.00)	−0.658	0.511
High educational level	57.9%	57.0%	58.8%	0.336	0.562
PIR	3.47 (3.16)	3.68 (3.09)	3.32 (3.27)	−1.515	0.130
Smoking status				35.435	<0.001
Never	47.2%	38.9%	56.8%		
Former	37.4%	44.6%	29.1%		
Current	15.4%	16.6%	14.1%		
Alcohol drinking	88.5%	93.2%	83.1%	73.269	<0.001
Never	11.3%	5.9%	17.6%		
Former	21.1%	21.9%	20.1%		
Mild	44.2%	51.2%	36.3%		
Moderate	12.8%	8.0%	18.2%		
Heavy	10.6%	13.6%	7.8%		
Physical activity, MET-min/week	3,710.00 (5,527.50)	4,030.00 (6,055.00)	3,150.00 (4,983.80)	−3.327	0.001
BMI, kg/m^2^	27.37 (6.38)	27.77 (5.83)	26.91 (7.06)	−3.286	0.001
BFM, kg	27.00 (11.50)	25.21 (10.63)	28.98 (11.29)	−6.918	<0.001
Systolic pressure, mmHg	125.00 (25.00)	124.00 (22.00)	126.00 (29.00)	−2.168	0.030
Diastolic pressure, mmHg	73.00 (15.00)	74.00 (15.00)	73.00 (15.00)	−1.627	0.104
ABPI	1.10 (0.15)	1.13 (0.13)	1.08 (0.13)	−6.915	<0.001
HbA1c, %	5.40 (0.50)	5.50 (0.50)	5.40 (0.50)	−2.741	0.006
Glucose, mmol/L	5.22 (0.84)	5.38 (0.88)	5.12 (0.78)	−7.336	<0.001
Creatinine, umol/L	79.56 (17.68)	88.40 (17.68)	70.72 (17.68)	−19.199	<0.001
Urea nitrogen, mmol/L	5.00 (2.14)	5.36 (2.15)	4.64 (2.14)	−5.957	<0.001
Triglycerides, mmol/L	1.26 (0.96)	1.33 (0.97)	1.21 (0.90)	−2.608	0.009
Total cholesterol, mmol/L	5.30 (1.31)	5.20 (1.27)	5.43 (1.27)	−4.415	<0.001
HDL-cholesterol, mmol/L	1.34 (0.55)	1.19 (0.39)	1.55 (0.62)	−14.034	<0.001
LDL-cholesterol, mmol/L	3.18 (1.14)	3.21 (1.18)	3.15 (1.11)	−0.774	0.439
CRP, mg/dl	0.21 (0.33)	0.17 (0.28)	0.25 (0.37)	−4.594	<0.001
Diabetes mellitus	13.6%	16.6%	10.2%	8.886	0.003
Hypertension	54.2%	52.4%	56.4%	1.637	0.201
Hyperlipidemia	80.9%	80.4%	81.6%	0.230	0.632
Use of antihypertensive drugs	23.0%	21.7%	24.4%	1.027	0.311
Use of antidiabetic drugs	7.4%	9.3%	5.3%	5.890	0.015
Use of lipid lowering drugs	20.1%	21.7%	18.2%	2.000	0.157
Total energy intake, kcal	1,908.00 (1,057.00)	2,191.00 (1,203.00)	1,641.00 (804.00)	−11.279	<0.001
Zinc, mg	9.86 (7.72)	12.26 (8.40)	8.29 (6.00)	−8.761	<0.001
Selenium, µg	94.90 (61.20)	109.20 (67.20)	80.95 (51.10)	−9.545	<0.001
Vitamin A, µg	490.00 (497.00)	508.00 (498.00)	461.00 (503.00)	−1.176	0.239
Vitamin C, mg	61.60 (99.80)	60.20 (99.70)	62.80 (98.90)	−0.566	0.571
Vitamin E, mg	5.73 (5.08)	6.47 (5.40)	5.29 (4.65)	−4.426	<0.001
Carotenoids, µg	5,685.00 (11,258.00)	5,360.00 (11,254.00)	6,177.00 (11,651.00)	−1.528	0.127
CDAI	−0.35 (3.54)	−0.54 (3.19)	−0.19 (3.90)	−0.823	0.410
All-cause death	29.8%	33.9%	25.2%	9.356	0.002
Cardiovascular death	8.8%	10.9%	6.4%	6.667	0.010

**Figure 2:**
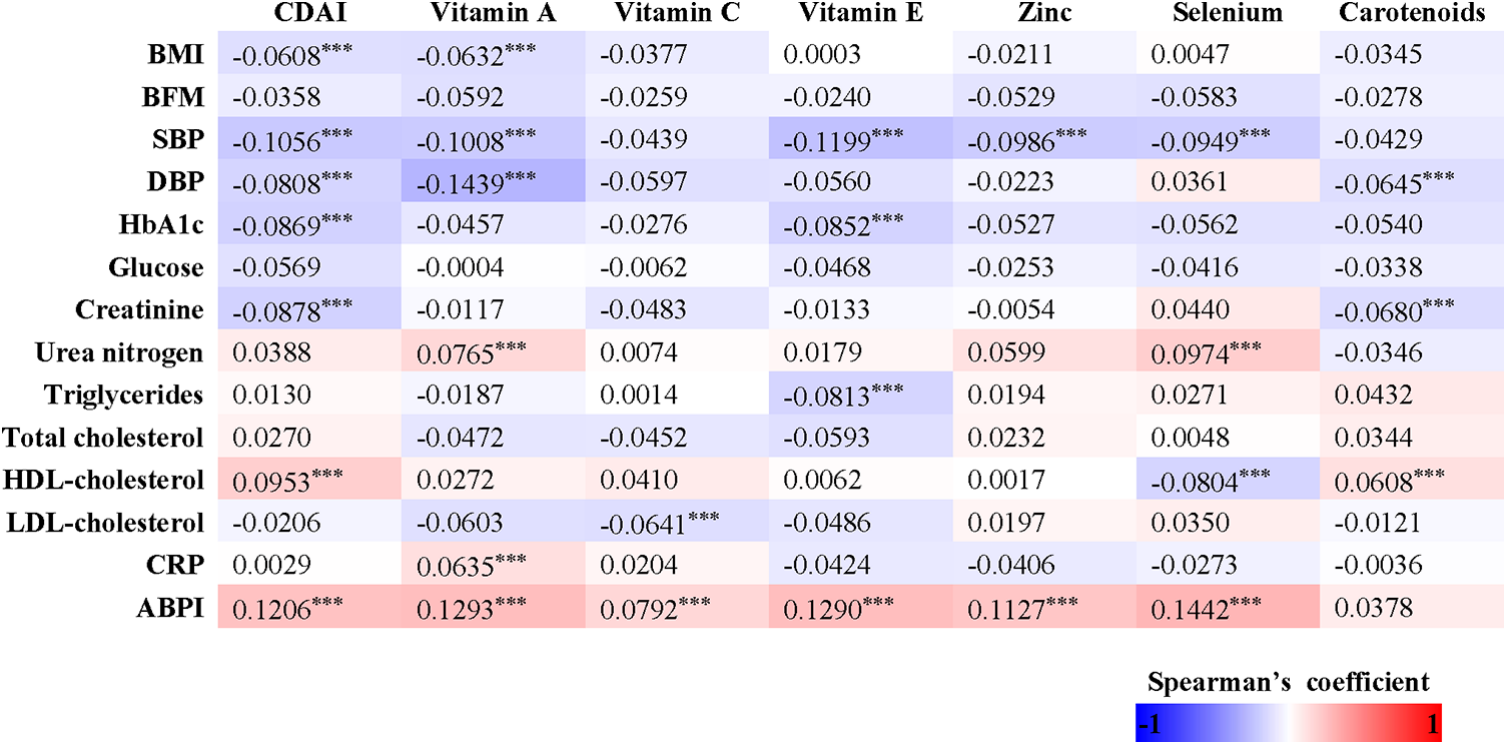
Heatmap representation of the spearman's correlation coefficients of dietary antioxidant intake and composite dietary antioxidant index with anthropometric measures (BMI, body mass index; and BFM, body fat mass), cardio-metabolic parameters (SBP, systolic blood pressure; DBP, diastolic blood pressure; glucose; HbA1c; creatinine; urea nitrogen; total cholesterol; triglycerides; LDL; HDL; CRP) and ABPI.

**Figure 3:**
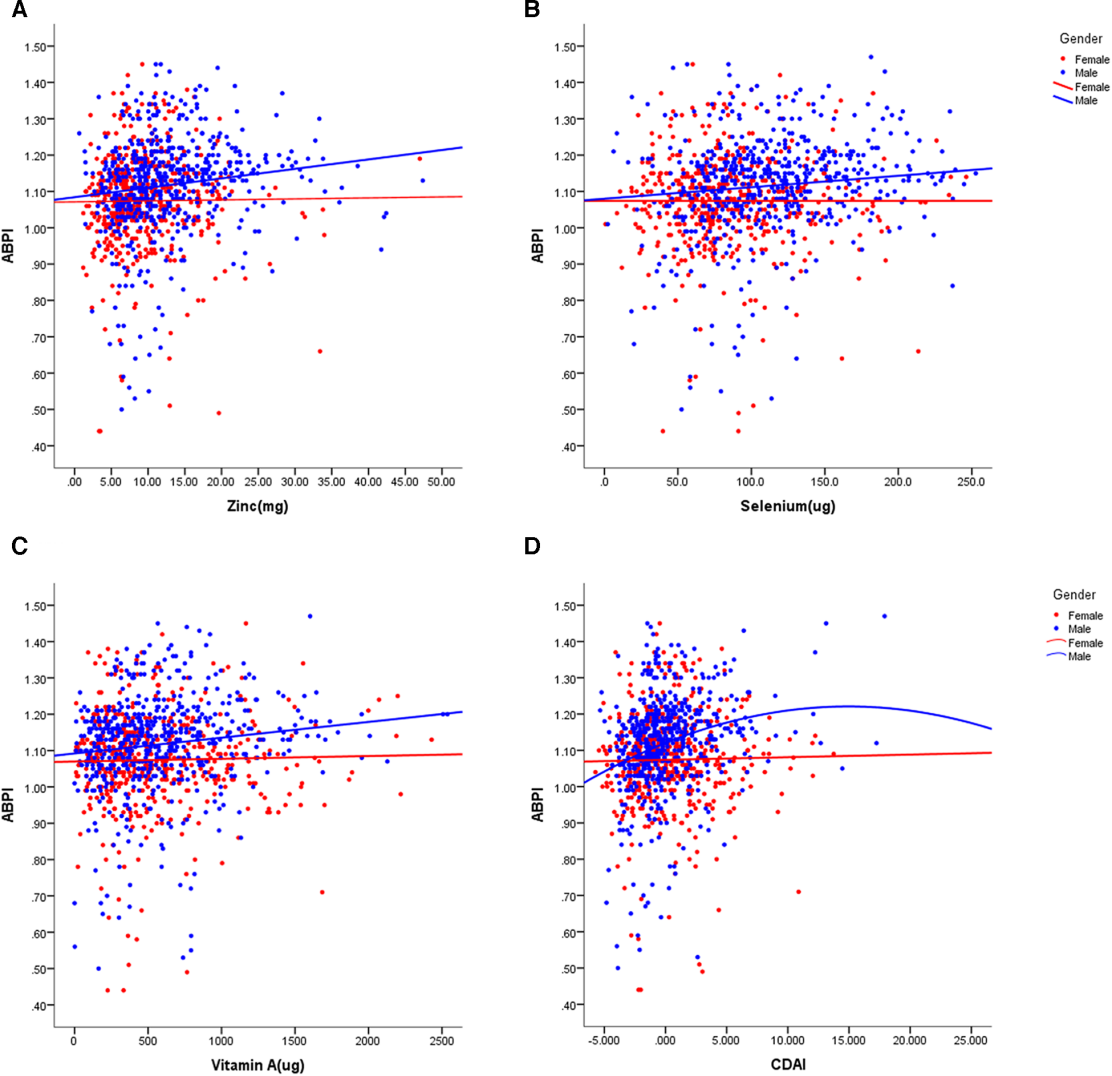
Scatter plots of the linear regression of dietary intake of zinc (**A**), selenium (**B**), vitamin A (**C**), and CDAI (**D**) with ABPI, stratified by gender. The models were adjusted for age, previous history and treatment of diabetes, hypertension and hyperlipidemia, PIR, educational level, smoking status, alcohol usage, physical activity, and total energy intake, anthropometric measures and cardio-metabolic parameters.

In our study, we observed that women had a decreased total energy intake compared to men, leading to a lower consumption of zinc (*p* < 0.001), selenium (*p* < 0.001), and vitamin E (*p* < 0.001), but not affecting the intake of vitamin A, vitamin C, and carotenoids. We investigated the relationships between dietary antioxidant intake, metabolic risk factors, and ABPI (Figure 2). Our findings revealed positive correlations between ABPI and the intake of zinc, selenium, vitamin A, vitamin C, and vitamin E. Additionally, we found significant interactions (*p* < 0.05) between the intake of these six antioxidants and gender, suggesting that their effects on ABPI varied between men and women. After adjusting for various factors such as age, medical history, and lifestyle factors, the association between higher ABPI in men and dietary intake of zinc, selenium, and vitamin A remained significant (Table 2 and Figure 3). Furthermore, when looking at quantile regression, the regression coefficients of these dietary antioxidants at different quantiles of ABPI increase as the quantile values increase. This indicates that as antioxidant intake increases, the impact on ABPI becomes greater (Table 3). In contrast, there were no significant associations observed between ABPI and dietary intakes of vitamin C, vitamin E, or carotenoids, whether considering the entire study cohort or when stratifying by gender.

**Table 2:** Hierarchical multiple regression between dietary antioxidant intake and ABPI.

Variates	Model 1		Model 2	
	Coefficients	Standardized coefficients	Coefficients	Standardized coefficients
Zinc	0.002*	0.106	0.001*	0.102
*R* ^2^	0.131		0.179	
F	5.858**		4.317**	
Δ*R*^2^	0.131		0.049	
ΔF	5.858**		2.441*	
Selenium	1.360 × 10^−4^	0.085	1.650 × 10^−4^*	0.103
*R* ^2^	0.113		0.162	
F	4.885**		3.776**	
Δ*R*^2^	0.113		0.050	
ΔF	4.885**		2.404*	
Vitamin A	3.307 × 10^−5^*	0.144	3.257 × 10^−5^*	0.142
*R* ^2^	0.140		0.188	
F	6.351**		4.585**	
Δ*R*^2^	0.140		0.048	
ΔF	6.351**		2.449*	

Model 1: The model was adjusted for age, previous history and treatment of diabetes, hypertension and hyperlipidemia, PIR, educational level, smoking status, alcohol usage, physical activity, and total energy intake.

Model 2: The model was adjusted based on model 1, incorporating anthropometric measures and cardio-metabolic parameters.

* *p* < 0.05.

** *p* < 0.001.

**Table 3:** Stratified regression analysis based on quartiles of the composite dietary antioxidant index in men and women using model 2.

Model 2	Character	Q1	Q2	Q3	Q4	*p* for trend	*p* for interaction
CDAI	Gender						0.001
	Male	ref	0.049 (0.018, 0.081)	0.056 (0.022, 0.091)	0.051 (0.012, 0.089)	0.019***	
	Female	ref	0.048 (0.013, 0.083)	0.037 (0.002, 0.071)	0.011 (−0.027, 0.049)	0.755	
Vitamin A	Gender						< 0.001
	Male	ref	0.043 (0.011, 0.074)	0.042 (0.009, 0.075)	0.049 (0.016, 0.082)	0.009***	
	Female	ref	−0.018 (−0.051, 0.015)	−0.017 (−0.051, 0.017)	−0.015 (−0.051, 0.021)	0.475	
Vitamin C	Gender						0.014
	Male	ref	0.029 (−0.002, 0.059)	0.045 (0.014, 0.076)	0.015 (−0.018, 0.047)	0.242	
	Female	ref	0.019 (−0.014, 0.053)	0.005 (−0.030, 0.040)	0.007 (−0.027, 0.042)	0.943	
Vitamin E	Gender						0.013
	Male	ref	0.023 (−0.011, 0.057)	0.019 (−0.015, 0.053)	0.028 (−0.010, 0.065)	0.224	
	Female	ref	−0.008 (−0.039, 0.024)	0 (−0.035, 0.036)	−0.006 (−0.046, 0.034)	0.896	
Zinc	Gender						< 0.001
	Male	ref	0.009 (−0.026, 0.044)	0.048 (0.012, 0.084)	0.053 (0.014, 0.092)	0.002***	
	Female	ref	0.039 (0.008, 0.070)	0.036 (0.001, 0.071)	0.011 (−0.032, 0.054)	0.447	
Selenium	Gender						< 0.001
	Male	ref	0.028 (−0.008, 0.064)	0.034 (−0.001, 0.070)	0.049 (0.010, 0.089)	0.020***	
	Female	ref	0.01 (−0.021, 0.041)	0.006 (−0.029, 0.041)	−0.004 (−0.047, 0.038)	0.917	
Carotenoids	Gender						0.019
	Male	ref	0.019 (−0.011, 0.049)	0.027 (−0.005, 0.058)	0.015 (−0.017, 0.046)	0.318	
	Female	ref	0.006 (−0.028, 0.040)	0.001 (−0.032, 0.033)	−0.007 (−0.042, 0.028)	0.618	

***p < 0.05.

Although women displayed lower dietary intake of certain antioxidants, our analysis of individual intake in relation to population norms revealed no significant difference between men and women in CDAI (*p* = 0.410). Overall, CDAI exhibited negative correlations with BMI, SBP, DBP, HbA1c, and creatinine, while showing a positive correlation with HDL-cholesterol (Figure 2). In a similar vein, we detected a noteworthy gender interaction in the association between CDAI and ABPI. This observation implies that CDAI may exert a significant impact on ABPI in men, while such an effect may not be as pronounced in women. Next, we conducted separate data analysis for males and females. For males, we performed adjustments for age, prior medical history, and management of diabetes, hypertension, and hyperlipidemia, in addition to factors such as personal income, educational attainment, smoking habits, alcohol consumption, physical activity, overall calorie intake, body measurements, and cardio-metabolic indicators (mode 2). Using quantile regression, our findings indicate that the regression coefficients of CDAI vary across different percentiles of ABPI. Specifically, these coefficients initially increase and then decrease with higher percentiles, suggesting the presence of a potential U-shaped association between CDAI and ABPI. Detailed results are presented in Table 3 and Figure 3.

**Figure 4:**
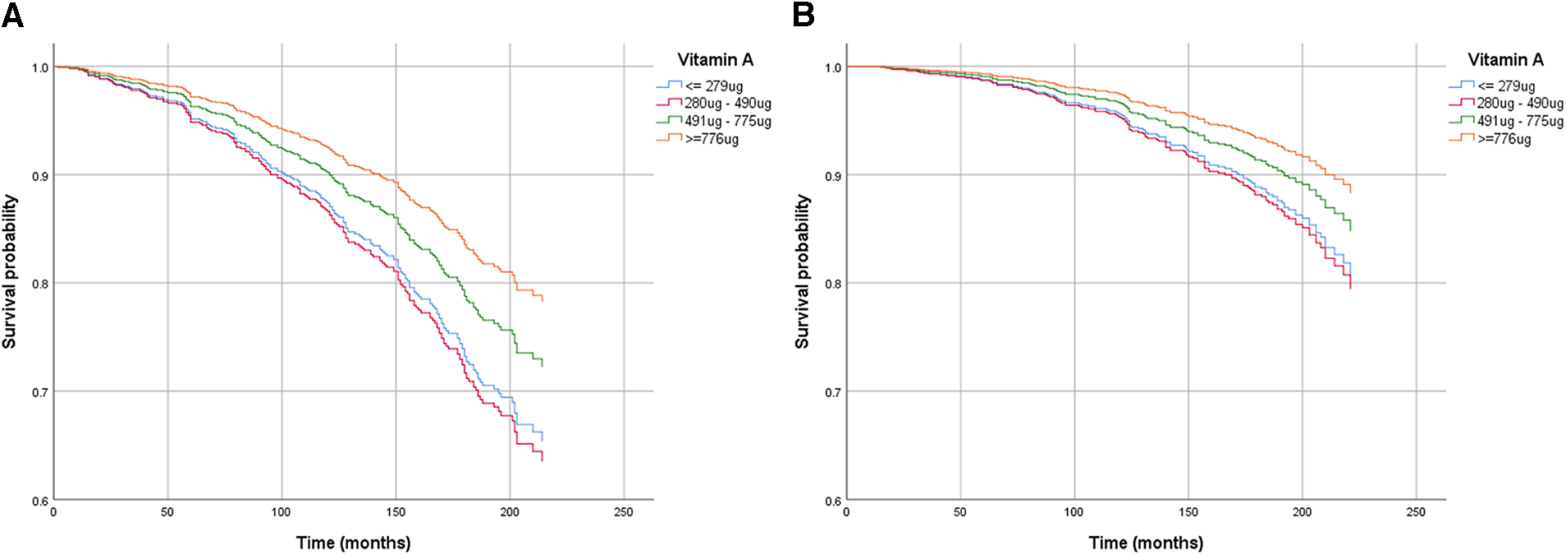
Cox regression of all-cause death mortality with vitamin A, stratified by gender [(**A**) for male, (**B**) for female]. The models were adjusted for age, previous history and treatment of diabetes, hypertension and hyperlipidemia, PIR, educational level, smoking status, alcohol usage, physical activity, and total energy intake, anthropometric measures and cardio-metabolic parameters.

Through mediation analysis, we found that the direct effects of CDAI, intake of Vitamin A, Vitamin C, and Vitamin E on all-cause death are statistically significant (*p* < 0.05). However, ABPI, serving as a mediator, does not statistically significantly mediate the indirect effects of antioxidant intake and CDAI on all-cause death (*p *> 0.05). Surprisingly, both the direct and indirect effects of antioxidant intake, CDAI, and ABPI on cardiovascular death are not statistically significant (Table 4). Moderation analysis shows a significant interaction between Vitamin A intake and gender in influencing all-cause death. Moderation analysis reveals a significant interaction between Vitamin A intake and gender in influencing all-cause death, while no significant interaction is observed between gender and other antioxidant intakes or CDAI (Table 5). Furthermore, the Cox regression of all-cause mortality with Vitamin A, stratified by gender, shows that a daily intake of Vitamin A more than 776 ug is especially beneficial for women (Figure 4).

**Table 4:** Mediation.

Path (X→M→Y)	Effects	Coefficients	95% CI	*p*-value
CDAI→ABPI→All-cause death	Total	−0.053	(−0.085, −0.020)	<0.001**
	Direct	−0.052	(−0.084, −0.020)	<0.001**
	Indirect	−0.001	(−0.003, 0)	0.250
CDAI→ABPI→Cardiovascular death	Total	0.004	(−0.015, 0.020)	0.510
	Direct	0.004	(−0.015, 0.020)	0.520
	Indirect	0	(−0.001, 0)	0.680
Vitamin A→ABPI→All-cause death	Total	−0.040	(−0.066, −0.010)	0.002*
	Direct	−0.039	(−0.065, −0.010)	0.002*
	Indirect	−0.001	(−0.003, 0)	0.296
Vitamin A→ABPI→Cardiovascular death	Total	0.005	(−0.011, 0.020)	0.410
	Direct	0.005	(−0.011, 0.020)	0.430
	Indirect	0	(−0.001, 0)	0.670
Vitamin C→ABPI→All-cause death	Total	−0.038	(−0.063, −0.010)	0.002*
	Direct	−0.037	(−0.062, −0.010)	0.002*
	Indirect	−0.001	(−0.002, 0)	0.414
Vitamin C→ABPI→Cardiovascular death	Total	0.001	(−0.018, 0.010)	0.810
	Direct	0.001	(−0.018,0.010)	0.820
	Indirect	0	(0, 0)	0.740
Vitamin E→ABPI→All-cause death	Total	−0.070	(−0.096, −0.040)	<0.001**
	Direct	−0.070	(−0.096, −0.040)	<0.001**
	Indirect	0	(−0.002, 0)	0.620
Vitamin E→ABPI→Cardiovascular death	Total	−8.090 × 10^−3^	(−3.840 × 10^−2^, 0.010)	0.470
	Direct	−8.970 × 10^−3^	(−3.830 × 10^−2^, 0.010)	0.480
	Indirect	8.480 × 10^−5^	(−5.860 × 10^−4^, 0)	0.850
Zinc→ABPI→All-cause death	Total	−0.009	(−0.038, 0.020)	0.570
	Direct	−0.007	(−0.037, 0.020)	0.630
	Indirect	−0.001	(−0.004, 0)	0.150
Zinc→ABPI→Cardiovascular death	Total	0.009	(−0.008, 0.020)	0.240
	Direct	0.008	(−0.009, 0.020)	0.260
	Indirect	0	(−0.001, 0)	0.680
Selenium→ABPI→All-cause death	Total	−0.029	(−0.061, 0)	0.063
	Direct	−0.029	(−0.060, 0)	0.072
	Indirect	−0.001	(−0.003, 0)	0.336
Selenium→ABPI→Cardiovascular death	Total	0.002	(−0.020, 0.020)	0.750
	Direct	0.001	(−0.020, 0.020)	0.770
	Indirect	0	(−0.001, 0)	0.720
Carotenoids→ABPI→All-cause death	Total	−0.019	(−0.043, 0.010)	0.150
	Direct	−0.018	(−0.043, 0.010)	0.150
	Indirect	0	(−0.002, 0)	0.710
Carotenoids→ABPI→Cardiovascular death	Total	−5.680 × 10^−3^	(−2.820 × 10^−2^, 0.010)	0.600
	Direct	−5.740 × 10^−3^	(−2.820 × 10^−2^, 0.010)	0.600
	Indirect	5.200 × 10^−5^	(−5.930 × 10^−4^, 0)	0.890

X, independent variable; M, mediator variable; Y, dependent variable.

Model (M→Y): The model was adjusted for age, gender, previous history and treatment of diabetes, hypertension and hyperlipidemia, PIR, educational level, smoking status, alcohol usage, physical activity, and total energy intake, anthropometric measures and cardio-metabolic parameters.

Model (X + M→Y): The model was adjusted for Quartile of X, M, age, gender, previous history and treatment of diabetes, hypertension and hyperlipidemia, PIR, educational level, smoking status, alcohol usage, physical activity, and total energy intake, anthropometric measures and cardio-metabolic parameters.

* *p* < 0.05.

** *p* < 0.001.

**Table 5:** Moderation.

Interaction	Coefficients	SE	*t*	*p-*value
CDAI × gender	−5.438 × 10^−4^	6.989 × 10^−3^	−0.078	0.938
Vitamin A × gender	1.102 × 10^−4^	4.896 × 10^−5^	2.251	0.025*
Vitamin C × gender	−1.463 × 10^−4^	2.752 × 10^−4^	−0.532	0.595
Vitamin E × gender	1.570 × 10^−3^	4.987 × 10^−3^	0.315	0.753
Zinc × gender	2.546 × 10^−3^	2.122 × 10^−3^	1.200	0.230
Selenium × gender	2.505 × 10^−5^	3.875 × 10^−4^	0.065	0.948
Carotenoid × gender	2.026 × 10^−7^	1.905 × 10^−6^	0.106	0.915

X, independent variable; Moderator (M): gender; Y, all-cause death.

Model (X × M→Y): The model was adjusted for X, M, X × M, age, gender, previous history and treatment of diabetes, hypertension and hyperlipidemia, PIR, educational level, smoking status, alcohol usage, physical activity, and total energy intake, anthropometric measures and cardio-metabolic parameters.

* *p* < 0.05.

## 4 Discussion

The current study evaluated the association between antioxidant intake, both at individual and cumulative levels, and ABPI. We discovered that dietary zinc, selenium, and Vitamin A are positively linked to ABPI in men, but not in women. Additionally, at the cumulative level, we observed a U-shaped association between CDAI and ABPI. ABPI is a measure of blood pressure in the legs compared to the arms. ABPI is typically used to assess the health of the arteries in the legs and to diagnose Peripheral Artery Disease (PAD). PAD is a condition where the arteries in the legs become narrowed or blocked, causing pain, numbness, or problems with walking (14). ABPI is typically measured using Doppler ultrasound, which uses sound waves to measure blood flow through the arteries. A lower ABPI may indicate PAD or other leg artery disease, while a normal ABPI is typically greater than 0.9. Some studies have shown that ABPI is associated with an increased risk of cardiovascular events such as heart attack, stroke, and death. ABPI may be an independent risk factor for cardiovascular disease and can predict the risk of future cardiovascular events (15–18). In general, men may have slightly higher ABPI values than women (19). This is partly because men typically have larger body mass and muscle mass, which can potentially influence the measurement results. This difference is usually within the normal range and should not lead to significant clinical disparities. It's important to note that ABPI depends on various factors, including age, genetics, and physiological condition. However, there is still debate about whether traditional cardiovascular risk factors such as high blood pressure, high cholesterol levels, smoking, and diabetes have gender differences in their impact on ABPI. In our cohort, men exhibit higher BMI, a greater history of smoking, a higher history of alcohol consumption, and elevated levels of fasting glucose and triglycerides, yet they show a higher ABPI, age partially explains why. Gender differences in the prevalence and severity of atherosclerosis become more significant as people age (20, 21). The changes in female hormone levels may potentially accelerate the development of atherosclerosis after menopause. Hormone-related effects on vascular oxidative stress partially account for these differences (22). Furthermore, in our study, men also demonstrate higher intake of zinc, selenium, and vitamin E, despite there being no gender differences in CDAI. Interestingly, in our study, although men can normalize ABPI through the intake of antioxidants, this seems to be unrelated to the final all-cause death and cardiovascular death. In fact, with the same intake, women seem to benefit more, resulting in lower all-cause death.

An adequate dietary intake of antioxidants may contribute to the prevention of atherosclerosis. A prospective study involving 4,564 healthy adults indicated a negative correlation between dietary zinc intake and subclinical atherosclerosis as measured by carotid intima-media thickness (23). The benefits of zinc in the context of atherogenesis suggest the inhibition of both LDL-cholesterol oxidation and caspases that are involved in several apoptotic pathways (24). As for selenium, an essential trace element in the human body, it is closely associated with the development of atherosclerosis. On one hand, selenium deficiency affects the synthesis of selenium-containing enzymes, leading to an increase in oxidative stress levels within the body. On the other hand, selenium can participate in various signaling pathways, inhibiting vascular calcification (25–27). Another randomized clinical trial has shown that supplementation with vitamin E alone can significantly reduce the progression of atherosclerosis in men, but not in women (28), which is corroborated by our findings. Overall, men are more prone to myocardial infarction and heart failure than women. However, women experience an increased risk after menopause, suggesting a cardiovascular protective role of estrogen. The latter exerts its cardiac protection through its antioxidant effect (29). Besides estrogen, other mechanisms may also contribute to cardiovascular risk, as hormone replacement therapy has not reduced cardiovascular risk in postmenopausal women (30). It is worth noting that in our study, CRP levels were higher in women compared to men, which aligns with previous research on peripheral artery diseases (31), suggesting that oxidative stress and pro-inflammatory biomarkers may be higher in women than in men. A partial explanation for these differences may be the varying expression and/or activity of antioxidant enzymes (namely, superoxide dismutase, glutathione peroxidase, and NADPH oxidase) between men and women (29).

It is challenging to accurately determine the independent impact of individual antioxidants on atherosclerosis because the dietary intake of these nutrients is highly correlated with each other. While specific antioxidants may play a role in atherosclerosis, potential biological interactions between dietary antioxidants and other nutrients should be taken into consideration. Therefore, our study utilizes a dietary antioxidant index (CDAI) to assess the effects of a combination of dietary antioxidants on atherosclerosis measured by ABPI. We found a potential U-shaped, rather than linear, association between CDAI and ABPI in men, but this pattern was not observed in women. This finding implies the possibility of potential biological interactions among dietary antioxidants, which could either synergize or offset the independent effects of individual antioxidants on atherosclerosis. Jaouad Bouayed and Torsten Bohn (32) have stated that the administration of high doses of isolated antioxidants may carry potential toxicity risks, primarily due to their prooxidative effects when present at elevated concentrations. Furthermore, these antioxidants have the capacity to interact with the beneficial concentrations of Reactive Oxygen Species (ROS) that are typically found in physiological conditions, which are essential for optimal cellular function. As such, caution is warranted when considering high-dose supplementation of isolated antioxidants, as it may disrupt the delicate balance required for normal cellular functioning. Further research is now needed to understand the mechanistic basis of these gender differences in cardiovascular disease risk.

There are some limitations to consider in our study. Despite analyzing a large sample size, our study does not have enough evidence to fully explain why there are gender differences in the effects of dietary antioxidants on atherosclerosis. Although we took into account several variables, there is still a possibility of other unknown factors that may affect the intake, metabolism, and ABPI related to antioxidants. Thus, it is crucial to conduct more thorough and extensive prospective studies on a larger scale to evaluate the impact of dietary antioxidant intake on the progression of atherosclerosis.

In conclusion, the findings from our research suggest that dietary antioxidants may have a gender-specific role in preventing arterial lesions and cardiovascular events and influence the outcome. However, further investigation is necessary to better comprehend the underlying mechanisms responsible for the gender disparities in the progression of atherosclerosis.

## Data Availability

The raw data supporting the conclusions of this article will be made available by the authors, without undue reservation.
